# The sorafenib anti-relapse effect after alloHSCT is associated with heightened alloreactivity and accumulation of CD8+PD-1+ (CD279+) lymphocytes in marrow

**DOI:** 10.1371/journal.pone.0190525

**Published:** 2018-01-05

**Authors:** Andrzej Lange, Emilia Jaskula, Janusz Lange, Grzegorz Dworacki, Dorota Nowak, Aleksandra Simiczyjew, Monika Mordak-Domagala, Mariola Sedzimirska

**Affiliations:** 1 L. Hirszfeld Institute of Immunology and Experimental Therapy, Polish Academy of Sciences, Wroclaw, Poland; 2 Lower Silesian Center for Cellular Transplantation with National Bone Marrow Donor Registry, Wroclaw, Poland; 3 Department of Immunology, Poznan University of Medical Sciences, Poznan, Poland; 4 Department of Cell Pathology, Faculty of Biotechnology, University of Wroclaw, Wroclaw, Poland; Wake Forest Institute for Regenerative Medicine, UNITED STATES

## Abstract

We studied three *FLT3* ITD acute myeloid leukemia (AML) patients who relapsed after allogeneic haematopoietic stem cell transplantation (alloHSCT) and received multikinase inhibitor (MKI) sorafenib as part of salvage therapy. MKI was given to block the effect of *FLT3* ITD mutation which powers proliferation of blast cells. However, the known facts that sorafenib is more effective in patents post alloHSCT suggested that this MKI can augment the immune system surveillance on leukaemia. In the present study, we investigated in depth the effect of sorafenib on the alloreactivity seen post-transplant including that on leukaemia. The patients (i) responded to the treatment with cessation of blasts which lasted 1, 17 and 42+ months, (ii) developed skin lesions with CD3+ cell invasion of the epidermis, (iii) had marrow infiltrated with CD8+ lymphocytes which co-expressed PD-1 (programmed cell death protein 1 receptor, CD279) in higher proportions than those in the blood (163±32 x10^3^ cells/μl vs 38±8 x10^3^ cells/μl, p<0.001). The Lymphoprep fraction of marrow cells investigated for the expression of genes involved in lymphocyte activation showed in the patients with long lasting complete remission (CR) a similar pattern characterized by (i) a low expression of *nitric oxide synthase 2* (*NOS2)* and *colony stimulating factor 2* (*CSF2)* as well as that of *angiopoietin-like 4 (ANGPTL4)* (supporting the immune response and anti-angiogenic) genes, and (ii) higher expression of *fibroblast growth factor 1* (*FGF1)* and *collagen type IV alpha 3 chain* (*COL4A3)* as well as *toll like receptor 9* (*TLR9)* and *interleukin-12* (*IL-12)* (pro-inflammatory expression profile) genes as compared with the normal individual. The positive effect in one patient hardly justified the presence of unwanted effects (progressive chronic graft-versus-host disease (cGvHD) and avascular necrosis of the femur), which were in contrast negligible in the other patient. The anti-leukemic and unwanted effects of sorafenib do not rely on each other.

## Introduction

The successful use of kinase tyrosine inhibitors in the treatment of acute myeloid leukaemia (AML) patients has promoted interest in this field, and a number of similar drugs have entered the market. Most of these drugs broadly inhibit kinases that conduct signals from different receptors but share targeted molecular characteristics [1,2]. One of these multikinase inhibitors (MKIs), sorafenib, was originally intended to treat advanced renal cell carcinoma and hepatocellular carcinoma patients by blocking the serine threonine kinase RAF-1 in the RAF/MEK/ERK signalling cascade. While sorafenib is used with the aim of slowing the signal transduction originating from the vascular endothelial growth factor (VEGF) and platelet-derived growth factor (PDGF) receptor families [3–5], it also efficiently blocks constitutive activation of Fms-related tyrosine kinase 3 mutated via internal tandem duplication (*FLT3* ITD) [3,6,7]. Furthermore, sorafenib affects the PI3K/mTOR/AKT signalling pathways essential for the proliferation and clonal expansion of antigen-specific T cells [8–10] and angiogenesis [11]. Patients with AML with *FLT3* ITD (Fms-related tyrosine kinase 3 mutated via internal tandem duplication *FLT3* ITD) frequently relapse after alloHSCT [12,13]. A second transplant can be performed only in about 20% of relapsing patients because of either poor biologic performance or the patient’s lack of willingness to undergo a second procedure [14]. It is postulated that sorafenib may be effective in the treatment of patients with relapse after alloHSCT but not when installed during upfront therapy [15]. The present study provides an analysis of the effect of sorafenib at the molecular and cellular level which documented prompt cessation of blasts and a long lasting effect associated with skewing of the gene expression profile toward gene-activated immune responsiveness and prolonged inflammation at the expense of genes involved in CD8 lymphocyte suppression and angiogenesis. Clinically it is reflected by an elevated blood level of von Willebrand factor and significant accumulation of CD8+CD279+ (PD-1 –programmed cell death protein 1 receptor) lymphocytes resembling phenotypically those described as tumor-infiltrating lymphocytes (TILs) in the marrow and GvHD in the skin and in a case-dependent manner in other organs. Study for the first time sheds a light on the mechanism of GvHD like lesion which appeared in about 40% of patients post alloHSCT on sorafenib[16]. This information is over great clinical value in the area of Nivolumab (anti-PD-1 (CD279) monoclonal antibody) therapy opening a new avenue for treatment of AML patients having over dismal prognosis *FLT3* ITD mutation and relapsing post HSCT [17].

## Patients

Patient UPN 952 (53-year-old man with *FLT3* ITD, *NPM1*-positive AML with a normal karyotype) received standard induction and consolidation therapy, and relapsed within three weeks. A course of ICE was given as a salvage and to consolidate the alloHSCT response, at which point his bone marrow was blast free. He relapsed 56 days after transplantation (S1A Fig).

Patient UPN 938 (50-year-old woman, *FLT3* ITD, *NPM1*-positive AML with normal karyotype) responded well to the induction and consolidation and was transplanted in complete remission (CR). She relapsed 33 weeks after the transplant (S1B Fig).

Both patients received transplants from unrelated donors (10/10 HLA alleles matched) on myeloablative conditioning and ATG (cumulative dose: 10 mg/kg body weight [b.w.]) and cyclosporine (CsA) as GvHD prophylaxis.

The third patient (UPN 1048, female, 50 years old, AML, *FLT3* ITD, *NPM1*-positive) completed the induction and consolidation therapy but relapsed within 3 months with skin involvement, FLAG therapy failed, then sequential intensified double step chemotherapy (clofarabine then busulfan and cyclophosphamide [BuCy]) was employed and transplanted from a haploidentical donor. Leukaemic blasts (70%) were seen in the marrow by the 30th day after HSCT (S1C Fig).

At post-alloHSCT relapse two patients received chemotherapy tailored to their biologic status the third one DLI and all sorafenib which dose started at 800 mg per day and then was tapered to 400 mg and 200 mg per day at a later time.

## Methods

*FLT3* ITD and *NPM1* mutations, PCR assays were performed using previously described primers and procedures [18,19]. The fluorescent dye-labelled PCR fragments were detected through capillary electrophoresis (ABI 3130, Life Technologies/Applied Biosystems, Foster City, CA).

Cytometry analysis of marrow and blood cell populations was performed using routine procedures [20] employing 4-colour flow cytometry (FACSCalibur, BD, Mountain View, CA) after staining with monoclonal antibodies (MoAbs) that recognized human CD45, CD8, CD279, or CD69 (all BD, Erembodegen, Belgium). Lymphocytes were analysed in a gate containing a population of a high-CD45/low-side-scatter cells.

Tissues biopsy analysis was performed with the use of a routine haematoxylin-eosin staining of paraffin blocks sections followed by immunostaining employing CD3, CD8, CD4, CD56, Ki67 and HLA DR monoclonal antibodies [21].

An *FLT3* Pathway Mutation PCR Array (SABiosciences, Qiagen) was used to determine the mutations at the checkpoints in the *FLT3*, *KRAS*, *HRAS*, *NRAS*, *MEK1*, *PIK3CA*, *BRAF* and *PTEN* genes according to the manufacturer’s protocol (S1 Appendix).

A panel of qPCR assays (Real Time Ready Custom Panels, Roche) was conducted in a LightCycler® 480 Instrument (Roche, Basel, Switzerland) to perform expression profiling of genes within the following categories: regulators of T-cell activation (*CD3D*, *CD3E*, *CD3G*, *CD7*, *CD80*, *CD86*, *CRTAM*, *CD8A*, *CD8B*, *CLEC7A*, *ICOSLG*, *IRF4*, *KIF13B*, *NCK1*, *NCK2*, and *PRLR*), regulators of Th1 and Th2 development (*CSF2*, *IFNB1*, *IFNG*, *IL12A*, *IL13*, *IL4*, *IL5*, *TLR2*, *TLR4 and TLR9*), T-cell proliferation (*IL10*, *IL12B*, and *SFTPD*), T-cell differentiation (*IL2*, *IL2RA*, *NOS2*, *SOCS5*, *SOCS3*, *and CD27*), Th1/Th2 differentiation (*HLA-DRA*, *IFNGR1*, *IFNGR2*, *IL12RB1*, *IL12RB2*, *IL18R1*, *IL2RA*, and *PVRL1*), T-cell polarization (*CD40LG*, *TGFB1*, *and CXCR4*), angiogenesis growth factors and receptors (*ANGPT1*, *ANGPT2*, *ANPEP*, *TYMP*, *EREG*, *FGF1*, *FGF2*, *FIGF*, *FLT1*, *JAG1*, *LAMA5*, *NRP1*, *NRP2*, *PGF*, *PLXDC1*, *STAB1*, *VEGFA*, and *VEGFC*), adhesion molecules (*KDR*, *PDGFRB*, *ANGPTL3*, *BAI1*, *COL4A3*, *and IL8*), proteases, inhibitors and other matrix proteins (*ANGPTL4*, *PECAM1*, *PF4*, *PROK2*, *SERPINF1*, and *TNFAIP2*), transcription factors (*HAND2* and *SPHK1*), growth factors and receptors (*FLT4* and *F12*) and blood coagulation agents (*F12*, *KNG1*, and *HRG*).

Total cellular RNA was isolated from the bone marrow after one-step density gradient centrifugation separation (Lymphoprep: d = 1.077 g/mL; Nycomed Pharma AS, Oslo, Norway) using the Direct-zol RNA MiniPrep kit (Zymo Research, Irvine, CA) according to the manufacturer’s protocol. RNA was quantified using a NanoDrop N D-1000 (Thermo Scientific, Waltham, MA) spectrophotometer. cDNA was then synthesized using the High Capacity cDNA Reverse Transcription Kit (Life Technologies, Foster City, CA) with a random hexamer primer, according to the manufacturer’s recommendations. Gene expression levels were normalized to those of the *HPRT1*, *RPL13A* and *RPLP0* housekeeping genes. The results were analysed using GenEx ver 6.1 (bioMCC, Freising, Germany).

### Western blotting for ERK and AKT detection

Samples of lysed cells from the blood after one-step density gradient centrifugation (Lymphoprep: d = 1.077 g/mL; Nycomed Pharma AS, Oslo, Norway) containing 30 μg of protein were denatured at 95°C for 10 minutes and processed with SDS-PAGE electrophoresis [22]. After the proteins were transferred to nitrocellulose membranes [23], they were stained with rabbit polyclonal antibodies directed against (1) AKT kinase (sc-8312) and its phosphorylated form (sc-13565, Santa Cruz Biotechnology, CA) or (2) ERK1/ERK2 (9102s) and phospho-Erk1/2 (Thr202/Tyr204) (9101s, Cell Signaling Technology, Danvers, MA). Then, goat anti-rabbit antibodies conjugated to horseradish peroxidase (HRP) were applied according to the manufacturer’s protocols. The immunoblots were developed using the Western blotting Luminol Reagent (Santa Cruz Biotechnology, CA). The blots were then scanned (ChemiDoc, Bio-Rad, Hercules, CA), and the levels of pERK and pAKT 1/2/3 were quantified using ImageLab software and normalized to ERK and AKT 1/2/3 expression levels, respectively.

The patients were clinically followed, which included (i) marrow trephine, (ii) skin and intestine biopsies with immunostaining for GvHD (EBMT and NIH guidelines) if clinically needed, (iii) marrow and blood cell cytometry covering the specificities required for leukaemia diagnosis, (iv) immune system cells analysis, and (v) CMV, EBV, and HHV6 DNA copies, as described elsewhere [24], while those of polyoma JC and BK were detected via RT PCR using the Altona Real-Time detection kit (RealStar BKV PCR Kit and RealStar JCV PCR Kit, Altona Diagnostics, Hamburg, Germany).

## Results

### Milestones of clinical observations

**Fig 1:**
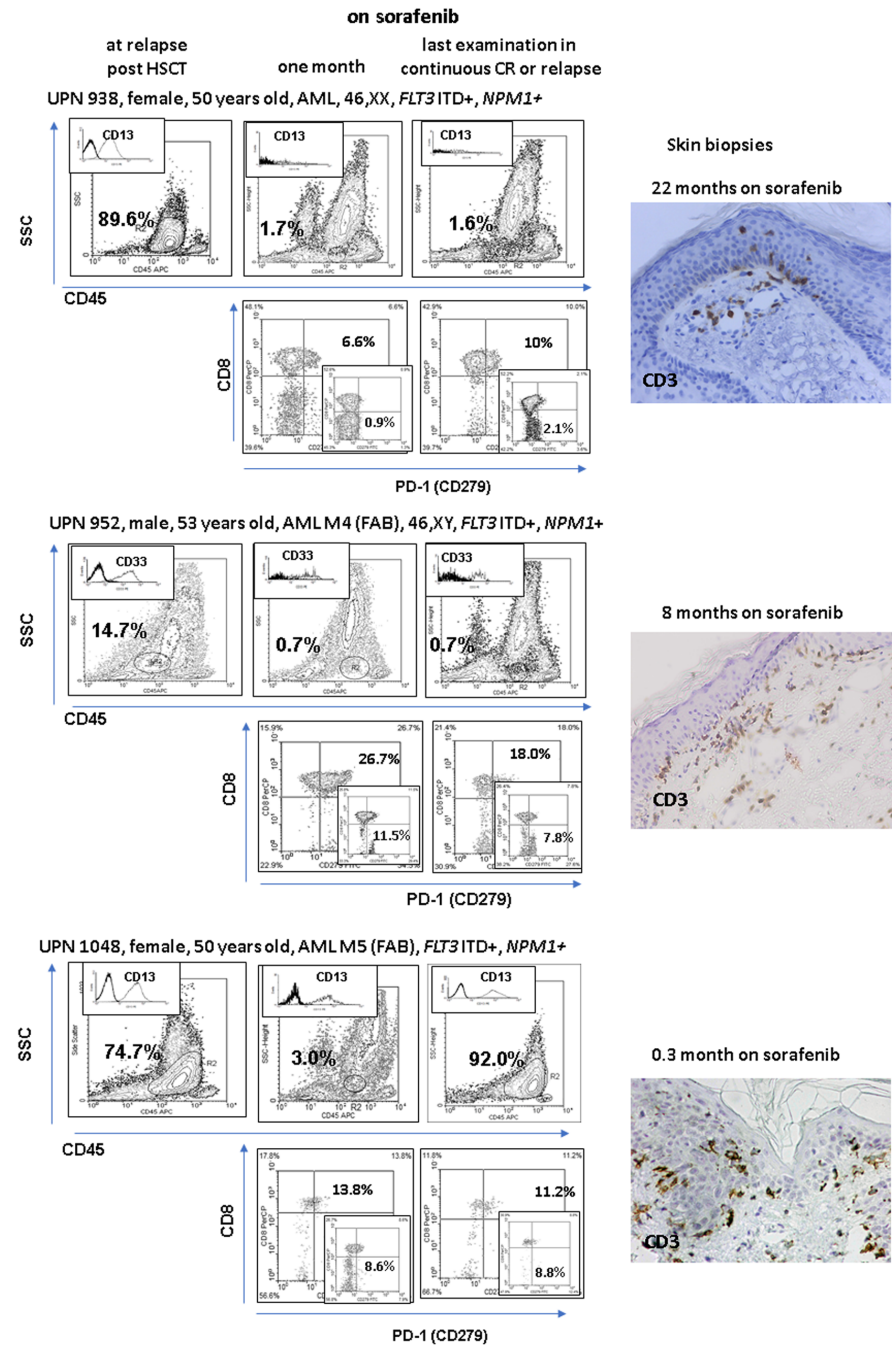
Follow-up of leukemic cells (CD45 dim, SSC low) in the patients on sorafenib (proportions of cells with a unique marker of leukaemia in the blasts gate are given in the upper left corner), in the lower rows PD-1 (programmed cell death protein 1 receptor, CD279) cell proportions in the marrow are shown and to compare the contour pictograms of the blood levels are depicted (lower right corner). Accumulation of activated T cells (CD69 positivity as a hallmark) in the marrow which become PD-1 positive witness the local at the marrow activation of T cells which become exhausted–a picture known from tumour-infiltrating lymphocyte studies [25]. The right-hand microphotographs illustrate the influx of lymphocytes in the skin biopsied in the area with maculopapular eruption (time of examination are shown within the figures). Please note that the positive response to sorafenib treatment in patients relapsing after HSCT is associated with the prevalence of PD-1 lymphocytes in the marrow and occurrence of an alloreactive reaction in the skin. However, the patients responding to the therapy display severe (UPN952) or only mild GvHD (UPN938) which was clinically apparent 22 months after the beginning of the treatment. Alloreactivity is not necessarily associated with a long-lasting response.

The presence of CR determined by phenotyping and the finding of eradication of *FLT3* ITD clones (PCR) was documented in all three patients and lasted one month, 16 months until the death in the GvHD patient and 42+ months in the other patient (Fig 1). During the observation period numbers of CD8+CD279+ (163±32 x10^3^ cells/μl vs 38±8 x10^3^ cells/μl, p<0.001), CD8+CD69+ (214±46x10^3^ cells/μl vs 12 ±2 x10^3^ cells/μl, p<0.001) as well as CD3+DR+ (694±144 x10^3^ cells/μl vs 991±129 x10^3^ cells/μl, p = 0.011) and CD56+ DR+ cells (109±18 x10^3^ cells/μl vs 156±20 x10^3^ cells/μl, p = 0.012) were higher in the marrow than in the blood at all checkpoints in all three patients.

Proportions of regulatory T (Treg) cells (CD4+CD25high+) in the blood were lower during the sorafenib treatment as compared to the values registered prior to that (0.13%±0.03% vs 0.36%±0.03%, p = 0.036).

**Fig 2:**
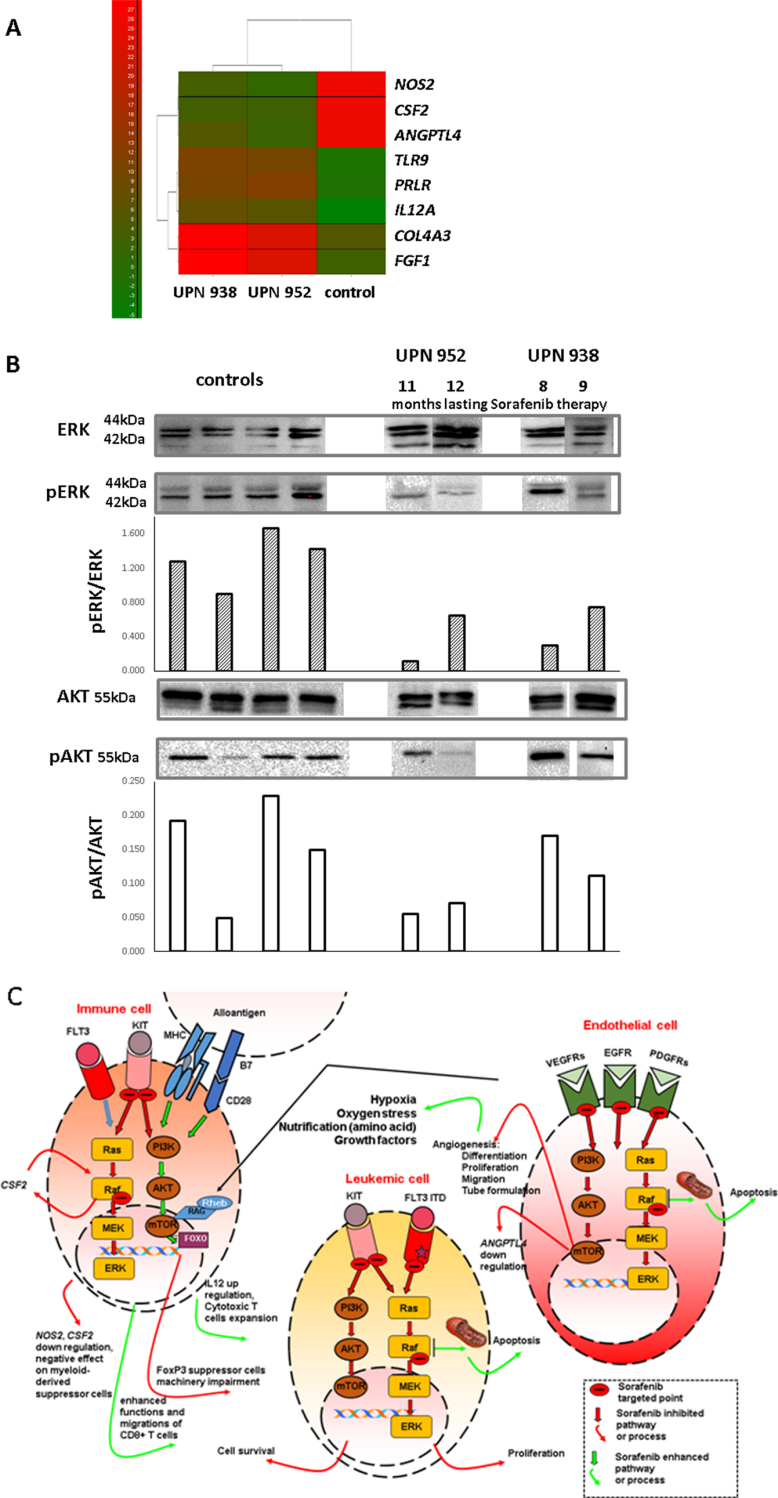
**A)** Heatmap of tested gene expression profiles from which 8 genes were selected with the highest numerical difference as compared to the control marrow values. A panel of qPCR assays (Real Time Ready Custom Panels, Roche) was conducted in a LightCycler 480 Instrument (Roche, Basel, Switzerland) to perform expression profiling of 88 genes involved in T cell activation, proliferation and differentiation, angiogenesis and blood coagulation. **B)** Western blot analysis of the level of ERK and AKT and its phosphorylated fraction in the blood mononuclear cells obtained on two occasions in two patients receiving sorafenib with or without maintenance chemotherapy. For comparison note the results of examination of 4 normal individuals (controls). Upper panel consists grouped images from different gels (please note dividing lines). The blots were scanned (ChemiDoc, Bio-Rad, Hercules, CA), and the levels of pERK and pAKT were quantified using ImageLab (Bio-Rad, Hercules, CA) software and normalized to ERK and AKT expression levels, respectively. **C)** Schematic illustration of the effect of sorafenib on the targeted kinases in the RAF/MEK/ERK and PI3K/mTOR/AKT signalling pathways considering the leukaemic and endothelial cells as well as immune cells as shown in this study. The targeted signal paths may be either blocked (red arrows) or enhanced (green arrows), which results in the clinically recognized symptoms listed appropriately. Leukaemic cells have mutated *FLT3* blocked as well as RAF kinase, reducing the cell proliferation and survival. The leukaemic cell burden is lowered and the remnant cells confront the T cells activated via the T cell receptor (TCR) with recognizable alloantigen [27]. Endothelial cells suffer from injury (VEGFR kinase is blocked), the tissue vasculature is poor, and the cells, including activated T cells, starve. The latter results in mTOR activation [8] with enhanced proliferation of T cells directly by enhancing the IL-12 [33] gene expression and indirectly due to the Treg cells’ impaired differentiation [29–31]. In addition, *NOS2* gene expression is downregulated, which hampers the function of myeloid-derived suppressor cells [34]. As a result, the graft-versus-leukaemia (GvL) effect can be instigated at the marrow level where the leukaemic cell alloantigens are recognized, and if TCR is triggered, local expansion is favoured by mTOR activation.

At the 9- and 11-month checkpoints, transcriptome profiling of 47 genes involved in lymphocyte activation and differentiation in the marrow cells enriched in lymphocytes was completed. Notably, a similar pattern of expression was found in two cases with a long lasting response, with higher expression levels of *TLR9*, *PRLR* and *IL12A* and lower expression of *NOS2* and *CSF2* (*GM-CSF*, Fig 2A) as compared to the expression levels seen in the marrow of a normal individual (male, 35 years old, with normal haematopoiesis). All of these genes were among the top 8 genes showing differential expression (low or high) as compared with the normal individual.

Among genes involved in angiogenesis and blood clotting, both analysed patients displayed upregulated *COL4A3* and *FGF1* and *IL-12* expression and downregulated *CSF2* and *ANGPTL4* (Fig 2A).

The activation statuses of ERK and AKT kinases in blood samples from 4 healthy controls and the patients on sorafenib at 8 and 9 (UPN 938) and 11 and 12 (UPN 952) months after installation of the therapy using Western blotting were analysed. Sorafenib effectively inhibited the phosphorylation of ERK but regarding AKT lower phosphorylation was only seen in the patient receiving a low dose rapamycin due to the progressive chronic GvHD (cGvHD, Fig 2B).

### The effect of sorafenib additive to its primary anti-leukaemic potential

In all patients skin alloreactivity was seen, which evolved to extensive cGvHD in one patient and it was mild and restricted to the skin of the cheeks or arms and thorax in the other two. Biopsy showed CD3+ (Fig 1) cells invading the epidermis. In all cases von Willebrand factor was elevated at the sorafenib treatment.

The cGvHD male patient’s symptoms included skin lesions (keratosis, skin atrophy), sicca syndrome and parenchymal hepatitis. The latter condition was aggravated by EBV reactivation; cGvHD symptoms were not alleviated by a standard steroid/mycophenolate mofetil regimen, and the patient received rapamycin (1 mg/day). Unwanted effects of sorafenib treatment, which included hand and foot syndrome and avascular femur necrosis, were observed 4 months after the MKI installation, while at the laboratory level, high von Willebrand factor activity (388%) was documented. The dose of sorafenib was tapered to 200 mg/day. The patient ultimately died of extensive cGvHD associated with herpes and polyoma virus reactivation in the presence of the unwanted effects of sorafenib treatment including avascular necrosis of the femur. He was free from leukaemia for 16 months, receiving sorafenib treatment as the only anti-leukaemic medication.

## Discussion

The anti-leukaemic potential of sorafenib in the present high-risk AML patients was associated with alloreactivity, which was increased but case dependent, being associated with a vigorous cGvHD in one patient but restricted to skin lesions in the other two. CD8+ cells infiltrating the marrow were in higher proportions CD279 positive as compared to the blood (Fig 1); thus they show the phenotype of tumour-infiltrating lymphocytes (TILs) [25]. The anti-angiogenic effect was seen as low expression of the *ANGPTL4* gene, whose level of expression is used to predict the response to sorafenib [26], and as a high level of von Willebrand factor indicating injury of the vessel endothelium. This effect was case dependent, from merely clinically observed symptoms up to avascular necrosis of the femur.

We specified the critical points along the RAF/MEK/ERK and PI3K/mTOR/AKT signalling pathways which are affected by MKI. *FLT3* ITD is targeted, which results in blast cessation. The effect on pERK kinase results in activation of cytotoxic T cells [27] and the lowering of *NOS2* expression, which is known to impair the function of myeloid derived suppressor cells, thus favouring anti-cancer surveillance [28]. Inhibition of Flt/VEGFR kinases makes the “vasculature bed” ineffective. This caused the avascular necrosis of the femur in one of our patients. Poor vasculature results in hypoxia and malnutrition of tissues, which creates a stressful situation for the cells, which is known to enhance the mTOR signalling [8]. Nutrient sensing in starved cells via mTOR favours the expansion and cytotoxicity of T cells and also adversely affects differentiation of FoxP3 regulatory cells [29–31] (Fig 2C). Indeed, sorafenib installation resulted in a decrease in the proportion of Treg cells in the blood.

The finding of the lowering of *CSF2* expression [32] and increased expression of the *IL-12* gene [33] in cells lends additional credit to the presented hypothesis, as these two features are known to be associated with sorafenib treatment (Fig 2A).

The new finding of our study is the observation of the accumulation of CD8+PD-1+(CD279+) lymphocytes (exhausted cells [25]) in the marrow. We hypothesize that the use of PD-1 antibody in leukaemic patients showing marrow accumulation of CD8+PD-1 lymphocytes may increase the potential of the immune system to fight leukaemia. The present observation also shows that inhibition of signalling pathways may affect both cancer cells and those involved in cancer surveillance, which should be considered and properly exploited if known and predictable.

## Ethic approval

The study was approved by the bioethics committee at the Medical University of Wroclaw (the opinion of the bioethics committee no. KB-369/2014).

## Supporting information

S1 Appendix *FLT3* Pathway Mutation PCR Array (SABiosciences, Qiagen) results. (XLSX)

S2 Appendix Sorafenib dosing and peripheral blood and bone marrow lymphocyte subpopulations (CD4+CD25high+, CD8+CD279+, CD8+CD69+, CD3+DR+, CD56+DR+) in the patients: UPN952, UPN938, UPN1048. Numeric data of the readings (described in the marker rows) as they were performed along the observation time; Please note that patients UPN 952 received sorafenib on alternate day regimen i.e. 400/200 mg. Each sheet represents each patient separately.
(XLSX)

S1 Fig The course and the outcome of the treatment of three patients (UPN 952, upper panel A; UPN 938, middle panel B, UPN1048, lower panel C) which relapsed after alloHSCT. The grey bar indicates sorafenib dosing, the triangles in the middle panel (UPN 938) indicate the courses of the maintenance therapy.
(TIF)
