# Correction: The sorafenib anti-relapse effect after alloHSCT is associated with heightened alloreactivity and accumulation of CD8+PD-1+ (CD279+) lymphocytes in marrow

**DOI:** 10.1371/journal.pone.0209108

**Published:** 2018-12-06

**Authors:** Andrzej Lange, Emilia Jaskula, Janusz Lange, Grzegorz Dworacki, Dorota Nowak, Aleksandra Simiczyjew, Monika Mordak-Domagala, Mariola Sedzimirska

The following information is missing from the Funding section: Publication of this study was supported by Wroclaw Centre of Biotechnology, programme The Leading National Research Centre (KNOW) for years 2014–2018.

## References

[1] Lange A, Jaskula E, Lange J, Dworacki G, Nowak D, Simiczyjew A, et al (2018) The sorafenib anti-relapse effect after alloHSCT is associated with heightened alloreactivity and accumulation of CD8+PD-1+ (CD279+) lymphocytes in marrow. PLoS ONE 13(1): e0190525 10.1371/journal.pone.0190525 29304116 PMC5755786

