# Supplementary figures and images for: The sorafenib anti-relapse effect after alloHSCT is associated with heightened alloreactivity and accumulation of CD8+PD-1+ (CD279+) lymphocytes in marrow
Source: PLoS One. 2018 Jan 5;13(1):e0190525. doi: 10.1371/journal.pone.0190525 (PMC5755786; doi:10.1371/journal.pone.0190525)

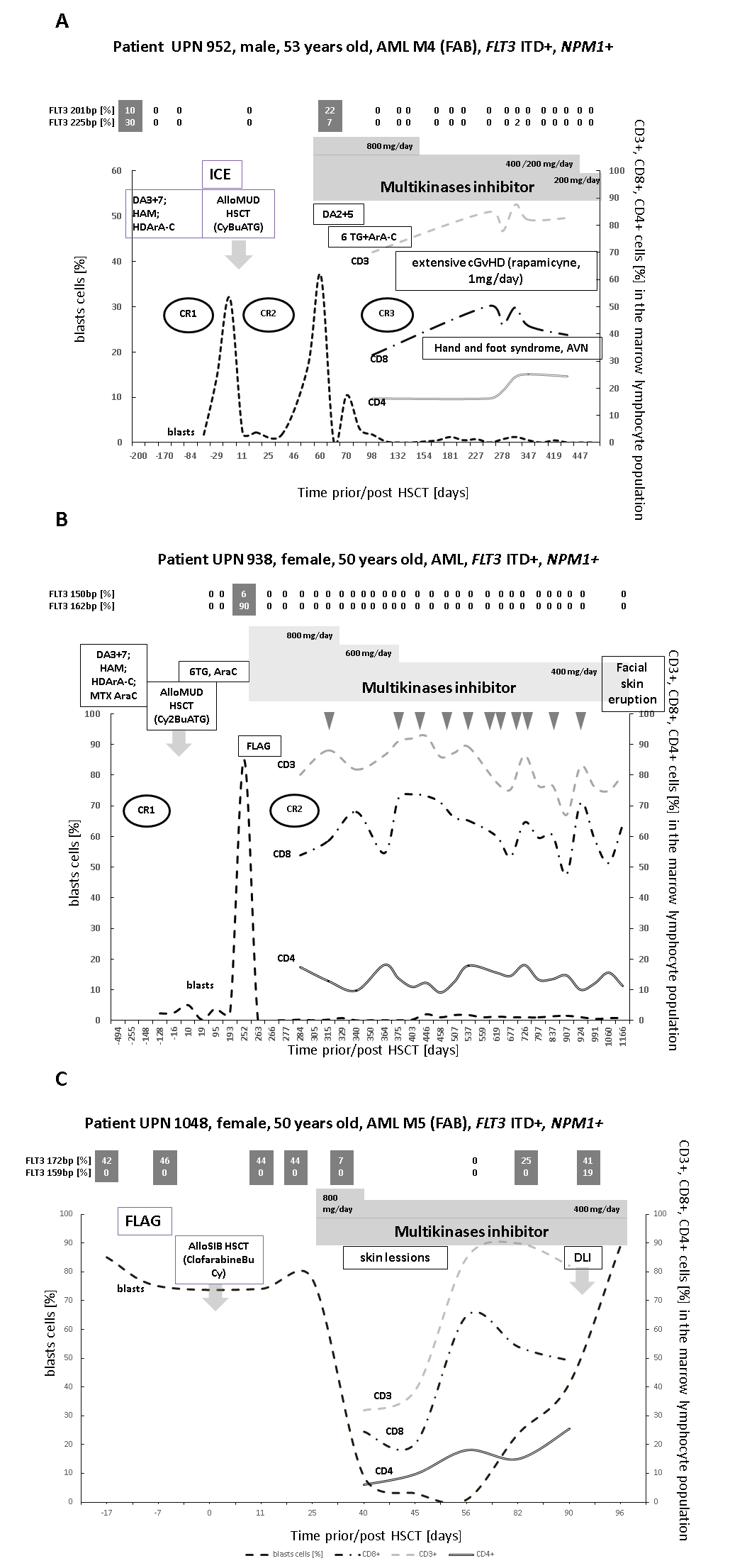

Supplement: S1 Fig — The grey bar indicates sorafenib dosing, the triangles in the middle panel (UPN 938) indicate the courses of the maintenance therapy. (TIF) [file pone.0190525.s003.tif]
